# Case report: Non-EBV associated cerebral vasculitis and cerebral hemorrhage in X-linked lymphoproliferative disease

**DOI:** 10.3389/fimmu.2024.1381472

**Published:** 2024-04-25

**Authors:** Bangtao Li, Weiming Chen, Xiaodi Cai, Yuanping Hai, Qiming Pang, Wei Xiang, Zhengzheng Zhang

**Affiliations:** ^1^Hainan Women and Children’s Medical Center, Pediatric Hospital, Fudan University, Haikou, China; ^2^Children’s Hospital of Fudan University, National Center for Children’s Medicine, Shanghai, China; ^3^Department of Endocrinology and Metabolism, Shunde Hospital, Southern Medical University, The First People’s Hospital of Shunde, Foshan, China; ^4^Key Laboratory of Tropical Diseases Prevention and Control, National Health Care Commission, Hainan Medical University, Haikou, China

**Keywords:** cerebral vasculitis, cerebral hemorrhage, X-linked lymphoproliferative disorders, SH2D1A gene, case report

## Abstract

X-linked lymphoproliferative disease (XLP) is a rare genetic disorder characterized by immune dysregulation. The three most common clinical phenotypes are EBV-associated infectious mononucleosis (FIM), abnormal gammaglobulinemia, and lymphoma. We present a rare case of XLP1 with neurovasculitis, which is non-EBV-related and involves multiple systems, a condition rarely seen in children. The patient initially presented with an unsteady gait, which progressively evolved into language and consciousness disorders. Additionally, CT scans revealed multiple nodules in the lungs. Subsequent genetic testing and brain tissue biopsy confirmed the diagnosis: XLP1-related cerebral vasculitis and cerebral hemorrhage. Tragically, during the diagnostic process, the child experienced a sudden cerebral hemorrhage and herniation, ultimately resulting in fatality. This case offers a comprehensive insight into XLP1-related cerebral vasculitis and cerebral hemorrhage, underscoring the significance of early diagnosis and prompt treatment, while also imparting valuable clinical experience and lessons to the medical community.

## Introduction

X-linked lymphoproliferative disease (XLP) is a rare and severe primary immunodeficiency disease caused by a mutation in the *SH2D1A* gene. The incidence of XLP is estimated to be between 1 in 1,000,000 and 3 in 1,000,000 (1). This disease has a poor prognosis, with 70% of XLP patients dying before the age of 10 and few surviving past the age of 40 if not treated by hematopoietic stem-cell transplant (HSCT). XLP has two recognized subtypes, namely XLP1 and XLP2. XLP1 is characterized by immune dysregulation triggered by the Epstein-Barr virus (EBV), leading to a condition called hemophagocytic lymphhistiocytosis (HLH). XLP1 is also associated with hypogammaglobulinemia (low levels of immunoglobulins) and malignant lymphoma. On the other hand, XLP2 is most commonly characterized by recurrent HLH and severe inflammatory bowel disease. Although rare, vasculitis can also occur in association with XLP. Here, we report an unusual clinical case of non-EBV-associated XLP presenting with cerebral vasculitis, cerebral hemorrhage, and multiple lung nodules. The patient was found to have a mutation in the *SH2D1A* gene (c.192G > A. p.W64X). This mutation is known to be associated with XLP1 and may contribute to the development of the observed clinical manifestations in this patient. The identification of this mutation highlights the importance of genetic testing in the diagnosis and management of XLP1.

## Case presentation

A 4-year and 5-month-old boy presented with a 10 day history of progressive unsteady gait, falls and tremors in the right limb. Over the 4 days prior to admission, his symptoms gradually worsened, manifesting as poor mental responsiveness, slow speech, occasional involuntary tremors of the jaw and limbs, without any signs of headache, fever, or seizures.

The child was G1P1, full-term vaginal delivery. He had a history of good health, with growth and development similar to his peers of the same age and gender. He was fully vaccinated according to Chinese standard vaccination program including receiving the BCG vaccination. There was no family history of abnormal miscarriages, prenatal abnormalities, stillbirths, or immunodeficiency on the maternal side.

Upon physical examination, the child was found to be alert, with decreased muscle tone in all four limbs. Muscle strength was rated as 4- in the upper limbs, 3+ in the right lower limb, and 3 in the left lower limb. Both knee-jerk reflexes were hyperactive, while the Babinski sign, Gordon sign, and Oppenheim’s sign were negative. Cardiovascular, respiratory, and abdominal examinations showed no abnormalities.

**Figure 1:**
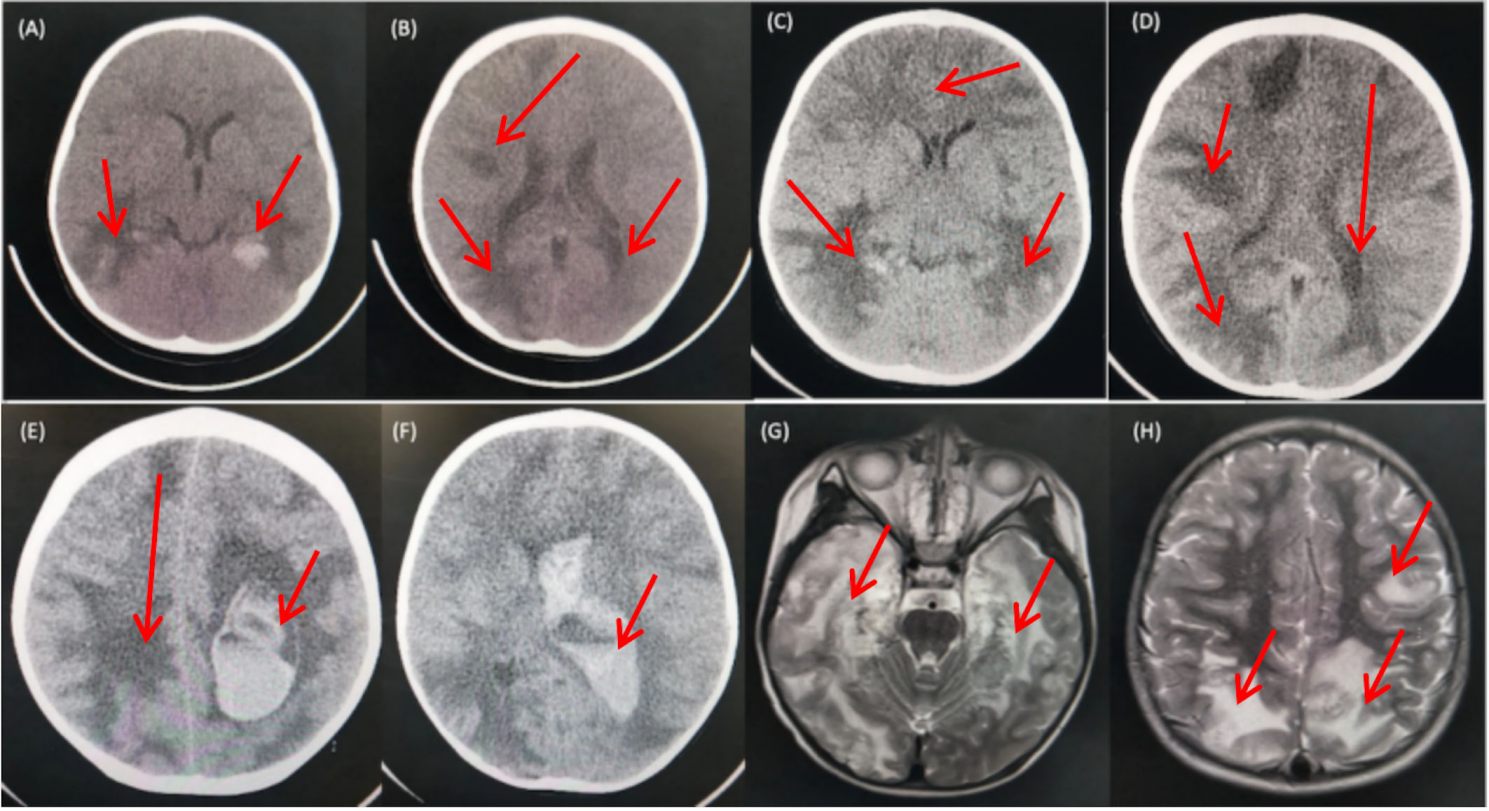
CT and MRI Findings of the Patient. **(A, B)** CT scans obtained prior to admission revealed multiple low-density patchy shadows in the bilateral frontal, parietal, temporal, and occipital lobes, with high-density patchy shadows observed in the bilateral temporal and occipital lobes. **(C, D)** CT scans obtained on day 16 of admission showed a decrease in density in the right occipital lobe and progressive involvement in the remaining lobes. **(E, F)** CT scans obtained on day 18 of admission revealed diffuse brain swelling, ventricular hemorrhage, subdural hemorrhage, and increased hemorrhage in the left parietal lobe. **(G, H)** MRI findings from the second day of admission demonstrated lesions with high intensities on T2-weighted images in the bilateral deep temporal and parietal lobes.

The cranial CT scan (**Figures 1A, B**) conducted on the day before admission revealed widespread multiple low-density lesions in bilateral frontal, parietal, temporal, and occipital lobes, suggestive of cerebral edema; high-density patchy shadows were observed in bilateral temporal and occipital lobes, indicating the possibility of hemorrhage. The cranial MR enhancement (**Figures 1G, H**) performed on the second day of admission showed multiple abnormal intracranial signals with abnormal enhancement. The possible diagnoses considered were acute necrotizing encephalitis, vasculitis with hemorrhage, or lymphoma. The chest CT scan showed multiple nodules in both lungs, while the whole abdomen CT scan showed normal findings.

Laboratory tests were detailed in **Table 1**, showing a significant decrease in immunoglobulins in the serum, suggesting the presence of hypogammaglobulinemia in the child; cerebrospinal fluid was clear and colorless, had elevated white blood cells, low glucose, and high protein levels, indicating the possibility of acute necrotizing encephalitis, vasculitis with hemorrhage, or lymphoma. Inflammatory markers evaluated during the course of the disease: white blood cells, neutrophils, C-reactive protein (CRP), procalcitonin (PCT), and erythrocyte sedimentation rate (ESR) remained relatively low throughout the disease course, with ferritin at 124 ng/ml (normal range 10-92 ng/ml). Interleukin-6 (IL-6) was elevated on the 10th day (51 pg/ml, normal range <10 pg/ml). In terms of etiology, the patient tested negative for serum antibodies to Toxoplasma gondii, rubella virus, cytomegalovirus, and herpes simplex virus 1/2. EB virus DNA and antibodies were negative in two separate tests. A single test for metagenomics next-Generation Sequencing (mNGS) in cerebrospinal fluid and bronchoalveolar lavage fluid was also negative. The nature of lung nodules was unclear, possibly inflammatory, tuberculosis, or tumorous. We planned to perform a lung biopsy to clarify the diagnosis and to exclude malignancy.

**Table 1:** Laboratory test report of the patient.

Laboratory indicators		Case (4.4 yrs)	Normal-range (age-matched)
*Day 1	WBC, ×10^9^/L	9.95	4-10
	Hb, g/dL	4.52	11-16
	N, ×10^9^/L	12.3	1.6-7.8
	PLT, ×10^9^/L	286	100-400
	CRP, mg/L	<8	<8
	PCT, ng/dL	0.07	<0.1
	blood sedimentation rate (ESR), mm/h	5	0-21
*Day 3	WBC, ×10^9^/L	1.96↓	4-10
	Hb, g/dL	12.3	11-16
	N, ×10^9^/L	0.15↓	1.6-7.8
	PLT, ×10^9^/L	312	100-400
	CRP, mg/L	66↑	<8
	PCT, ng/dL	0.9↑	<0.1
*Day 7	WBC, ×10^9^/L	2.08↓	4-10
	Hb, g/dL	12	11-16
	N, ×10^9^/L	0.61↓	1.6-7.8
	PLT, ×10^9^/L	230	100-400
*Day 17	WBC, ×10^9^/L	2.05↓	4-10
	Hb, g/dL	12	11-16
	N, ×10^9^/L	0.33↓	1.6-7.8
	PLT, ×10^9^/L	88↓	100-400
*Day 18	WBC, ×10^9^/L	1.96↓	4-10
	Hb, g/dL	9.3↓	11-16
	N, ×10^9^/L	0.13↓	1.6-7.8
	PLT, ×10^9^/L	96↓	100-400
*Day 19	WBC, ×10^9^/L	1.72↓	4-10
	Hb, g/dL	6.9↓	1.6-7.8
	N, ×10^9^/L	0.03↓	11-16
	PLT, ×10^9^/L	47↓	100-400
Biochemical indexes	Bilirubin, umol/L	6.8	3.4-17.1
	ALT, IU	11.4	8-71
	AST, IU	27.8	21-80
	Nitrogen (BUN), mmol/L	3	0.8-5.3
	Creatinine (Cr), umol/L	29	13-33
Coagulation function	Prothrombin Time(PT)	13.6	11-14.5
	International Normalised Ratio (INR)	1.02	0.8-1.2
	APTT, s	32.2	28-45
	Fibrinogen, g/L	2.41	2-4
Cerebrospinal fluid (CSF)	Color	yellow	colorless
	WBC,×10^6^/L	36↑	0-10
	Single nucleated cells	28/36	
	Protein, mg/L	26434.1↑	<450
	Glucose, mmol/L	2.0↓	2.5-4.4
	Chloride, mmol/L	124	120-132
Immunoglobulin (before IVIG)	IgG, g/L	0.5↓	4.95-12.74
	IgA, g/L	0.08↓	0.33-1.89
	IgM, g/L	0.3↓	0.65-2.01
Immunoglobulin (after IVIG)	IgG, g/L	19.5↑	4.95-12.74
	IgA, g/L	0.09↓	0.33-1.89
	IgM, g/L	0.38↓	0.65-2.01
Lymphocyte subsets	CD3+	68.98%	64%-73%
	CD3+ (absolute count)	793.8	
	CD4+	39.14↑ %	29%-36%
	CD4+(absolute count)	450.51	
	CD8+	27.01%	24%-34%
	CD8+(absolute count)	310.88	
	CD19+	23.05↑ %	14%-21%
	CD19+(absolute count)	265.25	
	CD16+CD56+	6.26↓ %	11%-23%
	CD16+CD56+(absolute count)	72.02	
	CD4+/CD8+	1.45	
	CD45+(absolute count)	1150.877	
Pathogens	EBV VCA-IgM, (serum)	negative	negative
	EBV VCA-IgG, (serum)	negative	negative
	EBV early antigen IgG, (serum)	negative	negative
	EBV nuclear antigen IgG, (serum)	negative	negative
	EBV PCR, (serum+CSF)	negative	negative
	Toxoplasma gondii-IgM, (serum)	negative	negative
	Toxoplasma gondii-IgG, (serum)	negative	negative
	Rubella virus-IgM, (serum)	negative	negative
	Rubella virus-IgG, (serum)	negative	negative
	Cytomegalovirus-IgM, (serum)	negative	negative
	Cytomegalovirus-IgG, (serum)	negative	negative
	Herpes simplex virus 1/2-IgM, (serum)	negative	negative
	Herpes simplex virus 1/2-IgG, (serum)	negative	negative
	mNGS(CSF+BLF)	negative	negative

yrs, years old; WBC, white blood cell; HB, hemoglobin; PLT, platelets; CRP, C-reactive protein; PCT, Procalcitonin; ALT, alanine aminotransferase; AST, aspartate aminotransferase; APTT, Activated Partial Thromboplastin Time(APTT); CSF, Cerebrospinal fluid; IVIG, intravenous immunoglobulin; EBV, Epstein-Barr virus; BLF,bronchoalveolar lavage fluid; *Days 1, 3, 7, 17, 18and 19 refer to days after hospital admission.

Initially, the child was treated with hemostasis medications (phenol sulfonamide, vitamin K1, Hemocoagulase Bothrops Atrox for Injection), cranial hypotension therapy (mannitol, glycerol fructose), and ceftriaxone sodium to combat infection. However, there was no improvement in the child’s condition during this period. Routine blood monitoring revealed a progressive decline in leukocytes and neutrophils, while the remaining blood parameters and coagulation were normal.

On the 3^rd^ day of admission, the child’s peripheral blood leukocyte count dropped to a minimum of 1.96 x 10^9^/L, and the absolute neutrophil count dropped to 0.15 x 10^9^/L. The child also had fever, rash, and elevated inflammatory markers, including a CRP level of 66 mg/L (normal rang <8 mg/L), and a PCT level of 0.9 ng/dL(normal rang <0.1 ng/dL). To address these issues, the patient was treated with linezolid for anti-infection purposes and received immunomodulatory dose immunoglobulin therapy (2 g/kg over 2 days), leading to a rise in IgG levels.

**Figure 2:**
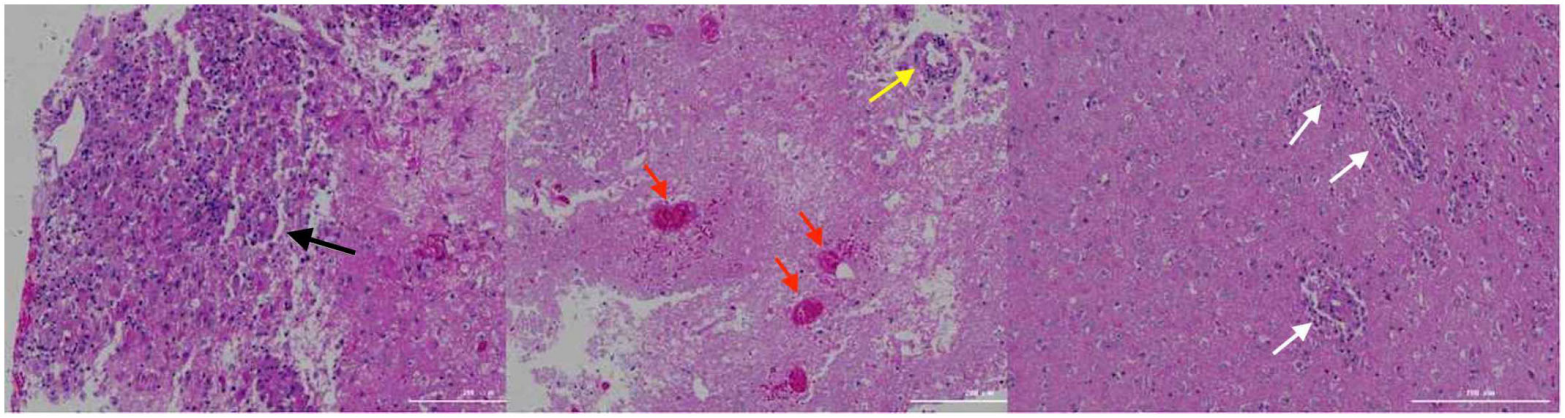
Brain tissue biopsy. The black arrows indicate lymphocytic infiltration with glial cell proliferation and eosinophilic changes; the yellow arrows indicate lymphocytic infiltration in the blood vessel walls; the red arrows indicate hemorrhage and necrosis; the white arrows indicate lymphocytic infiltration around the blood vessels.

On day 16^th^ day of admission, a repeat cranial CT scan was performed. The scan revealed that the right occipital lobe was less dense than before, while the rest of the brain showed progressive changes (**Figures 1C, D**). However, 8 hours before the scheduled lung biopsy, the child experienced sudden drooping of the left eyelid, difficulty in opening the eyes, and frequent vomiting. These symptoms were accompanied by two convulsive episodes occurring at 10-minute intervals, characterized by generalized seizures, double vision, tonic tremors of the limbs, an increased heart rate of 155 beats/min, transcutaneous oxygen saturation dropping to 90%, unequal pupils on both sides (6 mm on the left side and 2 mm on the right side), loss of light reflex on the left side, and a 2 mm right pupil with a sensitive reflex to light. Emergency blood gas and electrolyte tests showed normal results, and an emergency cranial CT scan was performed (**Figures 1E, F**). The scan revealed diffuse brain swelling, ventricular hemorrhage, subdural hemorrhage, and increased hemorrhage in the left parietal lobe. In response to these findings, the neurosurgeon performed craniocerebral hematoma removal, craniocerebral decompression, and a brain biopsy. Twenty-four hours after the surgery, the child remained in a deep coma with no spontaneous respiration. The right pupil measured 3 mm, and the light reflex was absent. The left eyelid was swollen, making it difficult to check the pupil. Blood pressure was maintained, and the child received invasive ventilator-assisted ventilation. Linezolid, combined with cefoperazone sodium sulbactam sodium, was administered to combat infection. Unfortunately, the prognosis was poor, and the family requested the withdrawal of all treatments and discharge. Tragically, the child passed away immediately after being discharged from the hospital. Two weeks after discharge, a biopsy of brain tissue revealed infectious encephalitis, characterized by a lymphocytic infiltration of the brain tissue and around blood vessels (see **Figure 2**). Two months later, a hemizygous variant in the *SH2D1A* gene was detected in the family’s whole exome sequence (**Supplementary Figure 1**, NM-002351: exon2: c.192G>A, p.W64X, inherited from the mother, validated by Sanger sequencing).

**Figure 3:**
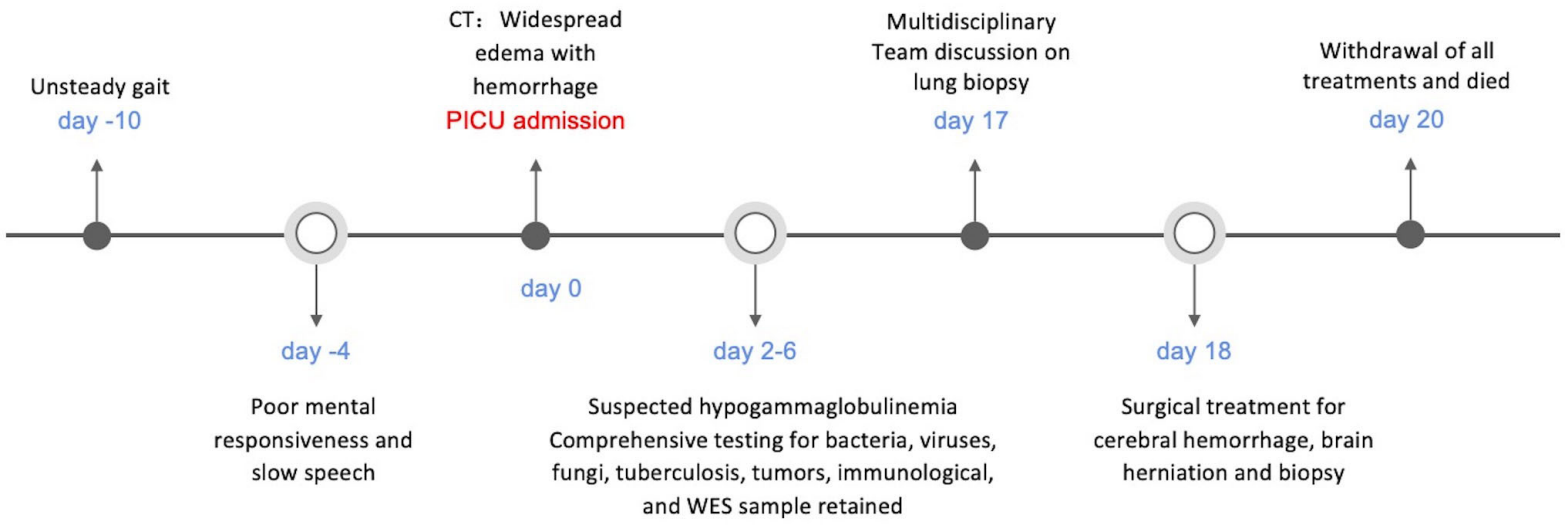
Timeline of case report.

It ultimately emerged that the child suffered from an X-linked lymphocytic abnormal proliferation disorder (see **Figure 3**). This disorder presented with multiple nodules, cerebral vasculitis, cerebral hemorrhage, and an immunodeficiency disorder, but without evidence of EBV infection or other infections, which is a rare occurrence. While lung nodules were not subjected to biopsy, the exclusion of potential causes such as fungal infections, tuberculosis, and connective tissue disorders led to the clinical inference of a plausible association with the primary disease.

## Discussion

We describe a rare occurrence of XLP1 with neurovasculitis, which, distinctively, was not associated with EBV and affects multiple bodily systems—a condition seldom observed in pediatric patients. The initial manifestation in this case was an unsteady gait, which gradually progressed to language and consciousness impairments. Through genetic testing, the patient was conclusively diagnosed with XLP1, pinpointing a mutation at the site c.192G>A in the *SH2D1A* gene. Both the child and his asymptomatic mother have a mutation in exon 2 of the *SH2D1A* gene, while the father has a normal genotype. These mutations result in abnormalities in the surface signaling lymphocyte activator-associated protein (SAP), which leads to impaired function of T and NK cells (2). The *SH2D1A* gene was first identified in 1998 in association with XLP, and more than 90% of XLP1 patients have mutations in this gene (3).

Vasculitis is a rare complication of XLP, with cerebral vasculitis being even rarer. In the past 20 years, there have been 12 reported cases (4–8) (**Supplementary Table 1**), with 3 of them associated with EBV infection. Central nervous system (CNS) vasculitis with onset of symptoms in the fCNS has multiple etiologies, making differential diagnosis crucial. Common causes include infections and autoimmune factors. CNS vasculitis related to XLP1 is difficult to diagnose early, often leading to delayed treatment. This case faced challenges in early diagnosis due to complex symptoms and involvement of multiple systems. Inconsistent results from various imaging and laboratory tests necessitated a comprehensive analysis of clinical manifestations and test results to establish a final diagnosis.

Although vasculitis is a rare complication of XLP1, CNS HLH and isolated CNS HLH are relatively common presenting features. The pathogenesis of CNS involvement in HLH is not yet clear, but it may be caused by a cytokine storm leading to disruption of the blood-brain barrier. CNS-HLH presents radiologically similar to neuroinflammatory diseases, with typical MRI findings of abnormal white matter signals around the ventricles, accompanied by volume loss and ventricular enlargement. Diffuse leptomeningeal and perivascular enhancement are commonly seen, with lesions frequently bilateral and nonspecific. Histopathologically, there is extensive tissue cell infiltration into the brain parenchyma, particularly in the white matter, leading to brain tissue atrophy and necrosis (9, 10) In this case there was no evidence of systemic HLH, brain histopathology was diagnostic of a lymphocytic vasculitis rather than demonstrating the non-specific features of CNS HLH, and imaging was not typical of CNS HLH. These findings all suggest a pathophysiology of vasculitis rather than the more typical HLH disease process seen in XLP.

Despite extensive investigations we were unable to identify an infectious or malignant cause of this patient’s disease. Although we were unable to attain a lung biopsy and tissue histopathology, we strongly suspect the same disease process identified in his brain was responsible for his pulmonary nodules.

HSCT is the best and only curative treatment for XLP1. Among the reported 12 cases of XLP1-related cerebral vasculitis, 3 patients underwent HSCT, with 2 showing clinical improvement and 1 dying. Among those who did not undergo HSCT, 1 was in a vegetative state, and the others died. Therefore, the mortality rate of XLP1-related cerebral vasculitis is high, and when conventional treatments are ineffective, early hematopoietic stem cell transplantation should be considered. Unfortunately, the disease progression in this case was rapid, and the child did not have the opportunity for HSCT treatment.

Therefore, when faced with clinically unexplained inflammation or lymphoproliferation, and extensive CNS damage in male children, it is crucial to consider primary immunodeficiency, and to thoroughly investigate the immune system. Early genetic testing for inborn errors of immunity in patients with atypical infectious and inflammatory complications, particularly where there is evidence of abnormal immune function, is essential and may lead to early consideration of HSCT and improved prognosis. Tissue diagnosis, even in cases where a more aggressive approach may be required, such as in the lung or brain biopsy, provides invaluable clues to an immunodeficiency diagnosis, and will reduce delays to diagnosis and treatment.

## Data availability statement

The datasets presented in this study can be found in online repositories. The names of the repository/repositories and accession number(s) can be found below: SCV004801966 (ClinVar).

## Ethics statement

The studies involving humans were approved by Ethics Committee of Children’s Hospital of Fudan University. The studies were conducted in accordance with the local legislation and institutional requirements. Written informed consent for participation in this study was provided by the participants’ legal guardians/next of kin. Written informed consent was obtained from the individual(s), and minor(s)’ legal guardian/next of kin, for the publication of any potentially identifiable images or data included in this article.

## Author contributions

BL: Data curation, Funding acquisition, Investigation, Resources, Writing – original draft. WC: Conceptualization, Formal analysis, Resources, Supervision, Writing – review & editing. XC: Data curation, Investigation, Methodology, Resources, Writing – review & editing. YH: Data curation, Formal analysis, Funding acquisition, Investigation, Resources, Writing – review & editing. QP: Data curation, Formal analysis, Funding acquisition, Investigation, Resources, Writing – review & editing. WX: Conceptualization, Data curation, Funding acquisition, Investigation, Methodology, Resources, Writing – review & editing. ZZ: Data curation, Formal analysis, Funding acquisition, Resources, Supervision, Writing – review & editing.

## Supplementary material

Supplementary Figure 1 Whole exome sequencing map of the family line. A missense mutation was identified at position 192 of the G base, resulting in a substitution from W to X at amino acid position 64.
