# Supplementary figures and images for: Case report: Non-EBV associated cerebral vasculitis and cerebral hemorrhage in X-linked lymphoproliferative disease
Source: Front Immunol. 2024 Apr 25;15:1381472. doi: 10.3389/fimmu.2024.1381472 (PMC11079196; doi:10.3389/fimmu.2024.1381472)

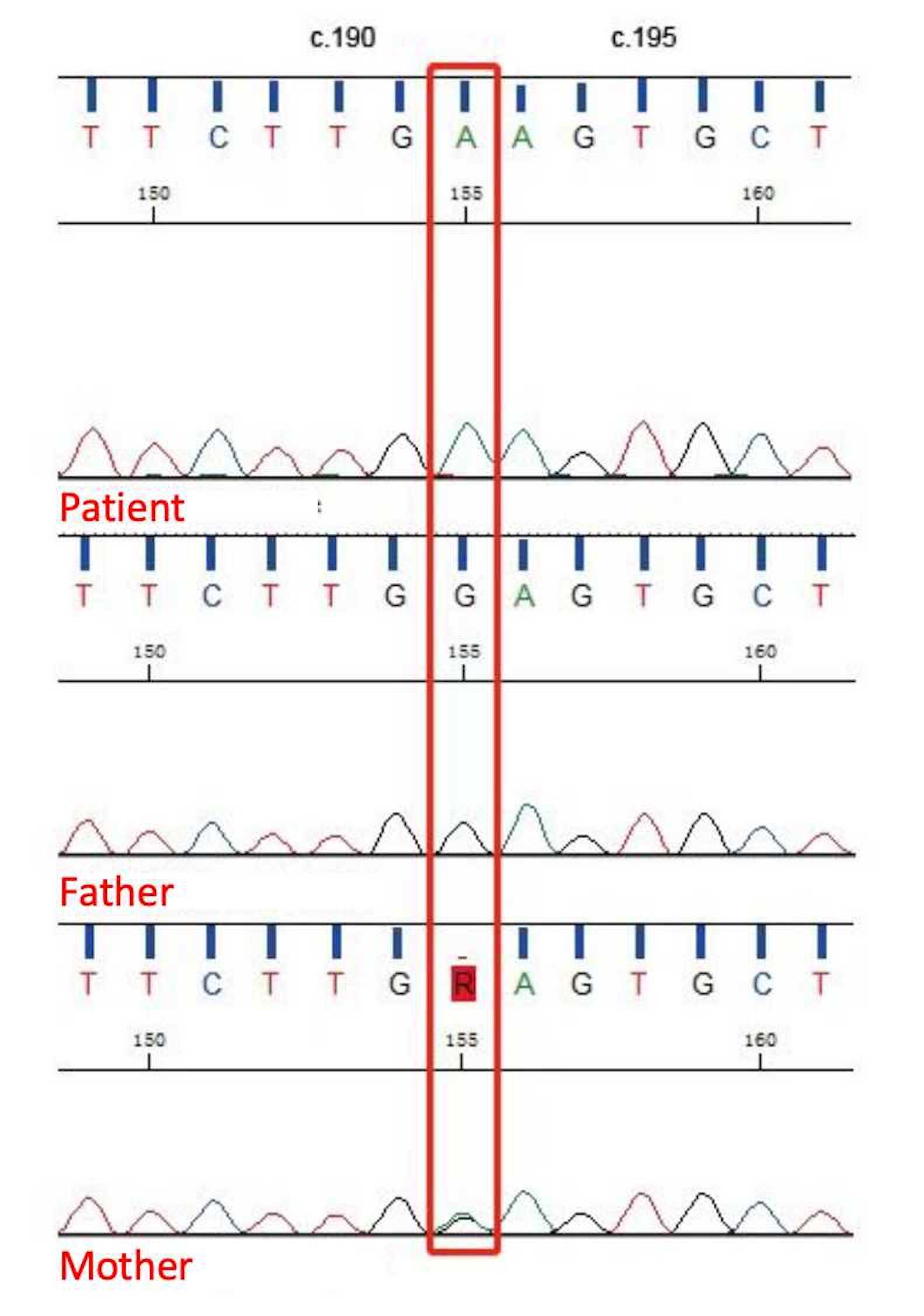

Supplement: Supplementary Figure 1 — Whole exome sequencing map of the family line. A missense mutation was identified at position 192 of the G base, resulting in a substitution from W to X at amino acid position 64. [file Image_1.jpeg]
